# Omapatrilat, an Angiotensin-Converting Enzyme and Neutral Endopeptidase Inhibitor, Attenuates Early Atherosclerosis in Diabetic and in Nondiabetic Low-Density Lipoprotein Receptor–Deficient Mice

**DOI:** 10.1080/15438600303728

**Published:** 2003

**Authors:** Zohar Levy, Ayana Dvir, Aviv Shaish, Svetlana Trestman, Hofit Cohen, Hana Levkovietz, Rita Rhachmani, Mordchai Ravid, Dror Harats

**Affiliations:** 1 Department of Medicine D Meir Hospital Kfar-Sava 44281 Israel; 2 The Institute of Atherosclerosis and Lipid Research Sheba Medical Center Tel-Hashomer Israel; 3 the Sackler Faculty of Medicine Tel-Aviv University Tel-Aviv Israel

## Abstract

Omapatrilat inhibits both angiotensin-converting enzyme (ACE) and neutral endopeptidase (NEP). ACE inhibitors have been shown to inhibit atherosclerosis in apoE-deficient mice and in several other animal models but failed in low-density lipoprotein (LDL) receptor– deficient mice despite effective inhibition of the reninangiotensin- aldosterone system. The aim of the present study was to examine the effect of omapatrilat on atherogenesis in diabetic and nondiabetic LDL receptor–deficient mice. LDL receptor–deficient male mice were randomly divided into 4 groups (n = 11 each). Diabetes was induced in 2 groups by low-dose STZ, the other 2 groups served as nondiabetic controls. Omapatrilat (70 mg/kg/day) was administered to one of the diabetic and to one of the nondiabetic groups. The diabetic and the nondiabetic mice were sacrificed after 3 and 5 weeks, respectively. The aortae were examined and the atherosclerotic plaque area was measured. The atherosclerotic plaque area was significantly smaller in the omapatrilat-treated mice, both diabetic and nondiabetic, as compared to nontreated controls. The mean plaque area of omapatrilattreated nondiabetic mice was 9357 ± 7293 *μ*m^2^, versus 71977 ± 34610 *μ*m^2^ in the nontreated mice (*P* = .002). In the diabetic animals, the plaque area was 8887 ± 5386 *μ*m^2^ and 23220 ± 10400 *μ*m^2^, respectively for treated and nontreated mice (*P* = .001). Plasma lipids were increased by omapatrilat: Meanplasma cholesterol in treated mice, diabetic and nondiabetic combined, was 39.31 ± 6.00 mmol/L, versus 33.12 ± 7.64 mmol/L in the nontreated animals (*P* = .008). The corresponding combined mean values of triglycerides were 4.83 ± 1.93 versus 3.00 ± 1.26 mmol/L (*P* = .02). Omapatrilat treatment did not affect weight or plasma glucose levels. Treatment with omapatrilat inhibits atherogenesis in diabetic as well as nondiabetic LDL receptor–deficient mice despite an increase in plasma lipids, suggesting a direct effect on the arterial wall.


*Experimental Diab. Res.*, 4:59–64, 2003
Copyright © 2003 Taylor & Francis
1543-8600/03 $12.00 + .00
DOI: 10.1080/15438600390214644

Taylor & Francis healthsciences

### Omapatrilat, an Angiotensin-Converting Enzyme and Neutral Endopeptidase Inhibitor, Attenuates Early Atherosclerosis in Diabetic and in Nondiabetic Low-Density Lipoprotein Receptor–Deficient Mice

**Zohar Levy,[1] Ayana Dvir,[1] Aviv Shaish,[2] Svetlana Trestman,[1] Hofit Cohen,[2] Hana Levkovietz,[2] Rita Rhachmani,[1] Mordchai Ravid,[1] and Dror Harats[2]**

[1]*Department of Medicine D, Meir Hospital, Kfar-Sava, Israel*
[2]*The Institute of Atherosclerosis and Lipid Research, Sheba Medical Center, Tel-Hashomer, Israel, and the Sackler Faculty of Medicine, Tel-Aviv University, Tel-Aviv, Israel*

**Omapatrilat inhibits both angiotensin-converting enzyme (ACE) and neutral endopeptidase (NEP). ACE inhibitors have been shown to inhibit atherosclerosis in apoE-deficient mice and in several other animal models but failed in low-density lipoprotein (LDL) receptor–deficient mice despite effective inhibition of the renin-angiotensin-aldosterone system. The aim of the present study was to examine the effect of omapatrilat on atherogenesis in diabetic and nondiabetic LDL receptor–deficient mice. LDL receptor–deficient male mice were randomly divided into 4 groups (n = 11 each). Diabetes was induced in 2 groups by low-dose STZ, the other 2 groups served as nondiabetic controls. Omapatrilat (70 mg/kg/day) was administered to one of the diabetic and to one of the nondiabetic groups. The diabetic and the nondiabetic mice were sacrificed after 3 and 5 weeks, respectively. The aortae were examined and the atherosclerotic plaque area was measured. The atherosclerotic plaque area was significantly smaller in the omapatrilat-treated mice, both diabetic and nondiabetic, as compared to nontreated controls. The mean plaque area of omapatrilat-treated nondiabetic mice was $9357 \pm 7293$ $\mu m^2$, versus $71977 \pm 34610$ $\mu m^2$ in the nontreated mice ($P = .002$). In the diabetic animals, the plaque area was $8887 \pm 5386$ $\mu m^2$ and $23220 \pm 10400$ $\mu m^2$, respectively for treated and nontreated mice ($P = .001$). Plasma lipids were increased by omapatrilat: Mean plasma cholesterol in treated mice, diabetic and nondiabetic combined, was $39.31 \pm 6.00$ mmol/L, versus $33.12 \pm 7.64$ mmol/L in the nontreated animals ($P = .008$). The corresponding combined mean values of triglycerides were $4.83 \pm 1.93$ versus $3.00 \pm 1.26$ mmol/L ($P = .02$). Omapatrilat treatment did not affect weight or plasma glucose levels. Treatment with omapatrilat inhibits atherogenesis in diabetic as well as nondiabetic LDL receptor–deficient mice despite an increase in plasma lipids, suggesting a direct effect on the arterial wall.**

**Keywords** Atherosclerosis; LDL Receptor–Deficient Mice; Omapatrilat

Vasopeptidase inhibition (VPI) involves simultaneous inhibition, with a single molecule, of 2 key enzymes: angiotensin-converting enzyme (ACE) and neutral endopeptidase (NEP) [1]. NEP has been reported to hydrolyze a number of endogenous vasoactive peptides, such as bradykinin and substance P, and is the principal enzyme degrading the natriuretic peptides [2]. By inhibition of the renin angiotensin aldosterone system (RAAS) and simultaneous potentiation of the natriuretic peptide system, VPIs reduce vasoconstriction and enhance vasodialation [1]. Recently, Arnal and colleagues [3] reported

Received 16 July 2002; accepted 12 January 2003.
The authors thank Asphir Ulman, MD, and Dan Belotherkovsky for technical assistance. Omapatrilat was kindly supplied by Bristol-Myers Squibb, Israel.
Address correspondence to Prof. M. Ravid, Department of Medicine, Meir Hospital, Kfar-Sava, 44281 Israel. E-mail: motirv@clalit.org.il that omapatrilat, a novel vasopeptidase inhibitor, prevented fatty streak deposition in apoE-deficient mice.

Angiotensin II was recently found to accelerate atherosclerosis in apoE-deficient mice and in low-density lipoprotein (LDL) receptor–deficient mice [4, 5]. ACE inhibitors reduce circulating levels of angiotensin II and were shown to inhibit LDL oxidation and retard atherosclerosis in apoE-deficient mice [6]. Atherosclerosis was attenuated by ACE inhibitors also in other experimental models of atherogenesis [7–13]. No effect could be demonstrated, however, in the LDL receptor–deficient mouse model [14] nor in the Watanabe heritable hyperlipidemic rabbit model [15].

The present study aimed to find whether omapatrilat would attenuate atherogenesis in diabetic (streptozotocin [STZ]-induced) and nondiabetic LDL receptor–deficient mice.

### METHODS

### Animals

LDL receptor–deficient mice with a C57BL/6 background were obtained from the Jackson Laboratory (Bar Harbor, Maine, USA) and bred in a local animal house (Sheba-Medical Center, Israel) on regular chow and water ad libitum. At 6 weeks of age, the mice were divided into 4 groups 14 mice each. In some of the animals, very low or very high lipid levels precluded their use. Finally, 11 animals were included in each group. Diabetes was induced in 2 groups using low-dose STZ. Upon confirmation of diabetes, the mice were fed Western-style diet consisting of 0.01% added cholesterol and 21% milk fat (TD98338; Harlan-Tekland, Madison, Wisconsin, USA). The animals were placed in a 12-hour light cycle and had ab libitum access to water. The animal experiments were performed according to National Institutes of Health (NIH) guidelines. The protocol was approved by the Sheba Medical Center Animal Subject Committee.

### Induction of Diabetes

To induce diabetes, STZ (Sigma), 50 mg/kg/day, in citrated buffer (pH = 7.4), was injected intraperitoneally for 5 consecutive days [16]. Glucose levels were determined in blood drawn from the retro-orbital plexus using Accutrend GC (Boeringer). The mice were considered diabetic if they had blood glucose levels higher than 250 mg/dL (15 mmol/L) at 2-hour fast. Omapatrilat, dissolved in water at a dose of 70 mg/kg/day was given using oral needle gavage. Control animals were gavaged with water.

### Cholesterol and Triglyceride Determination

Total 12-hour fasting plasma cholesterol and triglyceride levels were determined using an automated enzymatic technique (Boehringer-Mannheim, Germany). The blood was drawn from the retro-orbital plexus by a fine pipette with 10% EDTA.

### Tissue Preparation and Morphometric Analysis of Atherosclerotic Lesions

The heart was perfused from its apex with cold phosphate-buffered saline (PBS) via a canula placed in the left ventricle, with fluid drained from the right atrium. The hearts from the PBS-perfused mice were severed from the aorta at the base, embeded in OCT, and frozen. Every other section (10 $\mu$) throughout the aortic sinus was taken for analysis. Sections were evaluated for fatty streak lesions after staining with oil Red-O. Quantitation of atherosclerosis was performed with a high-resolution camera using computer-assisted image analysis, by an observer blinded to tissue identity.

### Statistical Analysis

The primary end point of the study was the atherosclerotic plaque area in the aorta. Based on a presumed 25% reduction of the plaque area by omapatrilate, 10 animals were needed in each group for a power of 0.8 and a type 1 error of 0.05. The data of the different groups were compared by 1-way analysis of variance and then by Student's *t* test for unpaired data using SigmaStat software. Data are expressed as mean ± SD. $P <$ .05 was accepted as statistically significant.

### RESULTS

Following the start of omapatrilat, the experiment lasted 5 weeks for the nondiabetic mice and 3 weeks for the diabetic mice. Four of the mice died. The first died immediately following an injection of STZ and the other three, one in each group, during the experiment. The omapatrilat-treated mice and the controls had a similar weight gain. At the beginning of the study, there were no significant differences in weight between the mice of the 4 groups. As expected, the STZ-treated mice gained significantly less weight than the nontreated mice (mean $0.05 \pm 2.4$ g versus $1.75 \pm 1.4$ g, respectively; $P =$ .009). Omapatrilat treatment had no effect on weight gain in either the diabetic or the nondiabetic mice (mean $-0.2 \pm 2.3$ g versus $0.3 \pm 2.5$ g, respectively, for treated and nontreated diabetic mice; and a mean of $1.4 \pm 1.7$ g versus $2.1 \pm 1.1$ g, respectively, for the nondiabetic mice) (Table 1).

### Plasma Glucose Levels

The STZ-treated mice had significantly higher plasma glucose levels than nontreated animals (mean $22.9 \pm 4.8$ mmol/L versus $6.9 \pm 0.9$ mmol/L, respectively; $P =$ .001). Plasma glucose levels were unaffected by omapatrilat in either the

**TABLE 1**
Body weight, plasma glucose, triglycerides, and cholesterol levels in omapatrilate-treated animals versus controls in diabetic and nondiabetic mice

| | Start (g) | Weight gain (g) |
|---|---|---|
| Body weight | | |
| Diabetes,[a] no treatment | 24.0 ± 4.4 | 0.3 ± 2.5 |
| Diabetes,[a] omapatrilat[b] | 22.3 ± 3.0 | −0.2 ± 2.3 |
| No diabetes, no treatment | 24.0 ± 4.4 | 2.1 ± 1.1 |
| No diabetes, omapatrilat[b] | 23.4 ± 3.7 | 1.4 ± 1.7 |
| | Start (mmol/L) | End (mmol/L) |
| Plasma glucose | | |
| Diabetes,[c] no treatment | 15.4 ± 4.1 | 22.8 ± 6.2 |
| Diabetes,[c] omapatrilat[d] | 16.8 ± 3.9 | 22.9 ± 2.9 |
| No diabetes, no treatment | 6.7 ± 1.2 | 7.0 ± 1.5 |
| No diabetes, omapatrilat[d] | 4.7 ± 1.4 | 6.8 ± 0.7 |
| Plasma triglyceride | | |
| Diabetes,[e] no treatment | 1.6 ± 0.5 | 10.8 ± 5.6 |
| Diabetes,[e] omapatrilat | 1.4 ± 0.6 | 14.5 ± 5.8 |
| No diabetes, no treatment | 1.5 ± 0.5 | 3.0 ± 1.3 |
| No diabetes, omapatrilat[f] | 2.1 ± 0.8 | 4.8 ± 1.9 |
| Plasma cholesterol | | |
| Diabetes,[g] no treatment | 5.1 ± 1.2 | 33.9 ± 8.2 |
| Diabetes,[g] omapatrilat[h] | 5.4 ± 0.9 | 39.5 ± 6.6 |
| No diabetes, no treatment | 5.0 ± 1.3 | 33.2 ± 7.4 |
| No diabetes, omapatrilat[h] | 5.5 ± 1.3 | 39.2 ± 5.7 |

[a]Diabetes induction resulted in a significantly lower weight gain ($P = .009$).
[b]Omapatrilat treatment did not affect weight gain.
[c]Diabetes induction resulted in a significantly higher plasma glucose leveles ($P = .001$).
[d]Omapatrilat treatment did not affect plasma glucose levels.
[e]Diabetes induction resulted in a significant increase in plama triglyceride levels ($P = .001$).
[f]Omapatrilat treatment resulted in a significant increase in plama triglyceride levels only among the nondiabetic mice ($P = .02$).
[g]Diabetes induction did not affect plasma cholesterol levels.
[h]Omapatrilat treatment resulted in a significant increase in plasma cholesterol levels ($P = .008$).

diabetic or the nondiabetic groups (mean 22.9 ± 2.9 mmol/L versus 22.8 ± 6.2 mmol/L, respectively, for the diabetic mice, $P = .49$; and a mean of 6.8 ± 0.7 mmol/L versus 7.0 ± 1.5 mmol/L, respectively, for the nondiabetic mice, $P = .77$) (Table 1).

### Plasma Lipids

The STZ diabetic mice had significantly higher plasma levels of triglycerides than the control mice (mean 11.9 ± 5.9 mmol/L versus 3.5 ± 1.8 mmol/L, respectively; $P = .001$). Omapatrilat treatment was associated with an increase in plasma levels of triglycerides in the diabetic as well as in the nondiabetic animals. The increase was statistically significant only in the non-diabetic group (mean 14.5 ± 5.8 mmol/L versus 10.8 ± 5.6 mmol/L, respectively, for the diabetic mice, $P = .16$; and a mean of 4.8 ± 1.9 mmol/L versus 3.0 ± 1.3 mmol/L, respectively, for the nondiabetic mice, $P = .022$) (Table 1, Figure 1). The induction of diabetes did not affect the plasma levels of cholesterol (mean 35.9 ± 8.0 mmol/L versus 36.1 ± 7.1 mmol/L, respectively, in the diabetic and nondiabetic mice). Following treatment with omapatrilat, there was a significant increase in plasma levels of cholesterol (a mean of 39.5 ± 6.6 mmol/L for the mice treated with omapatrilat versus 33.2 ± 7.4 mmol/L for the nontreated mice, $P = .008$). The rise in cholesterol was observed in both the diabetic and the nondiabetic animals (mean 39.5 ± 6.6 mmol/L versus 33.0 ± 8.2 mmol/L, respectively, for the diabetic mice, $P = .07$; and a mean of 39.2 ± 5.7 mmol/L versus 33.2 ± 7.4 mmol/L, respectively, for the nondiabetic mice, $P = .06$) (Figure 2).

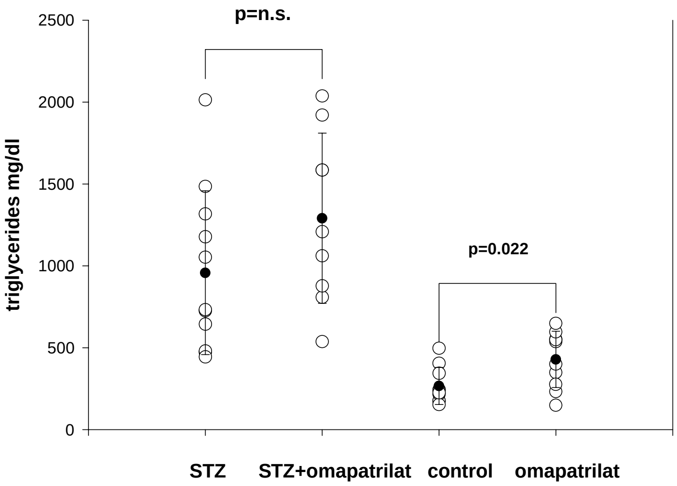

**FIGURE 1**
Plasma triglyceride levles. LDL receptor–deficient mice were divided into 4 groups (n = 11). Diabetes was induced in 2 groups using low-dose STZ, and 2 groups remained euglycemic. Omapatrilat was administered to one of the diabetic and one of the nondiabetic groups. Diabetes induced a significant rise in plasma triglycerides. Omapatrilat treatment resulted in a rise in plasma triglyceride level in both diabetic and nondiabetic mice.

### Atherosclerotic Plaque Area

Omapatrilat treatment was associated with a significant reduction of atherosclerotic plaque area in both the diabetic and the nondiabetic mice. In the diabetic mice treated with omapatrilat for 3 weeks, the mean aortic sinus plaques area was 8887 ± 5386 $\mu m^2$, as compared to a significantly greater

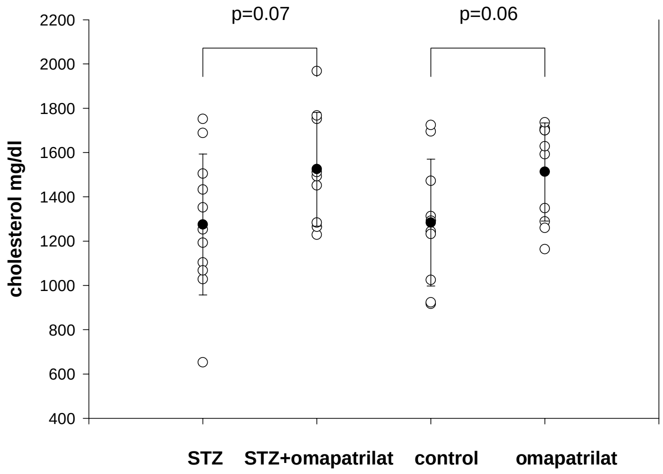

**FIGURE 2**
Plasma cholesterol levels. Diabetes induction did no change plasma cholesterol levels. Omapatrilat treatment resulted in a rise in plasma cholesterol level in both diabetic and nondiabetic mice.

area of $23220 \pm 10400\ \mu m^2$ in the nontreated animals ($P = .002$). A similar pattern was observed also in the nondiabetic mice, which were treated for 5 weeks, 2 weeks longer than the diabetic groups. The mean plaque area in the omapatrilat-treated mice was $9357 \pm 7293\ \mu m^2$, whereas in the nontreated mice, it measured $71977 \pm 34610\ \mu m^2$ ($P = .001$) (Figure 3).

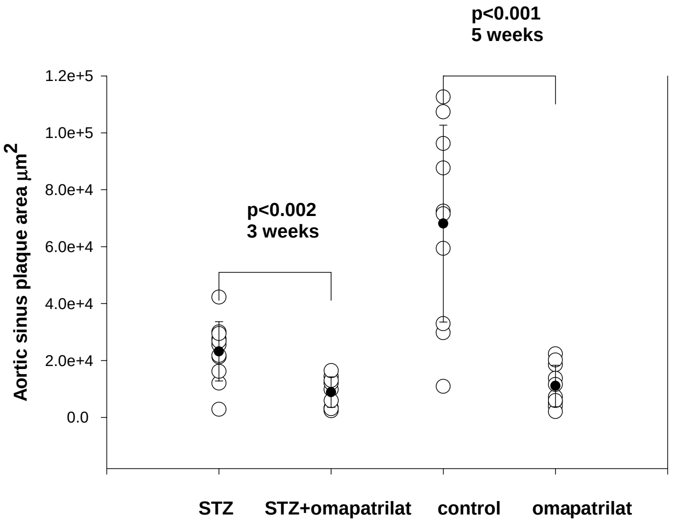

**FIGURE 3**
Aortic sinus plaque area. The experiment lasted 3 weeks for the diabetic mice and 5 weeks for the nondiabetic mice. Omapatrilat treatment induced a significant reduction of atherosclerosis in both diabetic and nondiabetic mice.

### DISCUSSION

The main finding of this study is that omapatrilat, a dual ACE and NEP inhibitor, retards atherogenesis in both diabetic and nondiabetic LDL receptor–deficient mice.

LDL receptor–deficient mice develop atherosclerotic lesions throughout the arterial tree and have been repeatedly used as a model of human atherosclerosis [17].

In this animal model, omapatrilat treatment induced no change in body weight or in plasma glucose levels in the diabetic as well as in the nondiabetic mice. Treatment with omapatrilat resulted, however, in an overall significant increase in plasma levels of cholesterol and triglycerides. The increase was statistically significant only in the nondiabetic mice, possibly due to the masking effect of diabetes on plasma triglyceride levels. A similar pattern was reported by Arnal and colleagues [3] in apoE-deficient mice treated with omapatrilat.

The decrease in atherosclerosis induced by omapatrilat in the diabetic as well as in the nondiabetic experimental animals despite a rise in plasma lipid levels is probably an expression of a direct effect on the arterial wall. The more pronounced atherosclerosis in the nondiabetic animals is born out by the longer period of exposure to the atherogenic diet. ACE inhibitors were repeatedly reported to counteract atherogenic stimuli in various experimental models of apoE-deficient mice, hamsters, rabbits, minipigs, and monkeys [8–13]. A specific NEP inhibitor, candoxatril was shown to attenuate atherosclerosis induced by high-cholesterol diet in rabbits [18], but failed to reproduce this effect in apoE-deficient mice [19].

The antiatherogenic activity of ACE inhibitors may be partially born out by reducing the availability of angiotensin II. This peptide has been repeatedly found to induce atherosclerosis in experimental models of apoE-deficient and LDL receptor–deficient mice [4, 5]. Angiotensin II may accelerate atherosclerosis by several mechanisms. Angiotensin II promotes the expression of vascular cellular adhesion molecule (VCAM)-1 [20] and of monocyte chemoattractant protein (MCP)-1 [21], stimulates the production of lipid-oxidizing reactive oxygen species [22], and induces apoptosis of endothelial cells [23]. Chobanian and colleagues [15] reported that the antiatherogenic effect of ACE inhibitors in the Watanabe heritable hyperlipidemic rabbit model was associated with the ability to lower blood pressure. A blood pressure lowering dose of the drug also inhibited atherogenesis. However, when the dose of the ACE inhibitor was sufficient to reduce serum and aortic ACE activity, but insufficient to lower blood pressure, atherosclerosis was not reduced. A similar pattern was observed with irbesartan, an angiotensin II receptor antagonist. At a low dose, irbesartan blocked the pressor effect of infused angiotensin II, but did not lower the blood pressure and atherosclerosis was not blocked. At higher doses, irbesartan lowered blood pressure and attenuated atherosclerosis [24]. Blood pressure was not measured in the present experiment. The duration of the experiment, 3 to 5 weeks, is, in any case, insufficient to explain reduction of atherosclerosis as secondary to vasodilatiation. Additional experiments are needed to clarify this issue. It was recently reported that ACE inhibitors failed to decrease atherosclerosis in LDL receptor–deficient mice, despite a significant inhibition of the renin-angiotensin aldosterone axis [14]. The dual effect of omapatrilat may induce endothelial changes more effectively than ACE inhibition alone, thus retarding atherogenesis where ACE inhibitors alone fail.

Whether the dual action of omapatrilat as an ACE inhibitor and a NEP inhibitor may be translated into a better clinical outcome is not clear. The results of the IMPRESS study, which compared omapatrilat with the ACE inhibitor lisinopril in patients with heart failure, suggest a trend in favor of omapatrilat on the combined end point of worsening heart failure and death [25]. The effect of omapatrilat as compared with ACE inhibition alone deserves further investigation both in animal and in human studies.

### REFERENCES

[1] John, C. B. (1999) Vasopeptidase inhibition: A new concept in blood pressure management. *J. Hypertens.,* **17**(Suppl. 1), S37–S43.

[2] Roques, B., Noble, F., Dague, V., Fournie-Zaliski, M., and Beaumont, A. (1993) Neutral endopeptidase 24.11: Structure, inhibition, and experimental and clinical pharmacology. *Pharmacol. Rev.,* **45,** 87–146.

[3] Arnal, J. F., Castano, C., Maupas, E., Mugniot, A., Darblade, B., Gourdy, P., Michel, J. B., and Bayard, F. (2001) Omapatrilat, a dual angiotensin-converting enzyme and neutral endopeptidase inhibitor, prevents fatty streak deposit in apolipoprotein E-deficient mice. *Atherosclerosis,* **155,** 291–295.

[4] Alan, D., Micheal, W. M., and Lissa, A. C. (2000) Angiotensin II promotes atherosclerotic lesions and aneurysms in apolipoprotein E-deficient mice. *J. Clin. Invest.,* **105,** 1605–1612.

[5] Daughtery, A., and Casis, L. A. (1999) Chronic angiotensin II infusion promotes atherogenesis in low-density lipoprotein receptor −/− mice. *Ann. N. Y. Acad. Sci.,* **892,** 108–118

[6] Hayek, T., Attias, J., Breslow, J., and Keidar, S. (1995) Antiatherosclerotic and antioxidative effects of ACE inhibitors in apolipoprotein E deficient mice. *Circulation,* **92,** 2998–3002.

[7] Hernandez-Presa, M., Bustos, C., Ortego, M., Tunon, J., Renedo, G., Ruiz-Ortega, M., and Edigo, J. (1997) Angiotensin-converting enzyme inhibition prevents arterial nuclear factor kappa B activation, monocyte chemoattraction protein-1 expression, and macrophage infiltration in a rabbit model of early accelerated atherosclerosis. *Circulation,* **95,** 1532–1541.

[8] Hoshida, S., Nishida, M., Yamashita, N., Igarashi, J, Aoki, K., Hori, M., Kuzuya, T., and Tada, M. (1997) Vascular angiotensin-converting enzyme activity in cholesterol-fed rabbits: Effects of enalapril. *Atherosclerosis,* **130,** 53–59.

[9] Aberg, G., and Ferrer, P. (1990) Effects of captopril on atherosclerosis in cynomolgus monkeys. *J. Cardiovasc. Pharmacol.,* **15**(Suppl.), S65–S72.

[10] Jacobson, L. S., Persson, K., Aberg, G., Andersson, R. G., Karlberg, B. E., and Olsson, A. G. (1994) Antiatherosclerotic effect of angiotensin-converting enzyme inhibitors captopril and fosinopril in hypercholesterolemic minipigs. *J. Cardiovasc. Pharmacol.,* **24,** 670–677.

[11] Kowala, M. C., Valentina, M., Recce, R., Beyer, S., Goller, N., Durham, S., and Aberg, G. (1998) Enhanced reduction of atherosclerosis in hamsters treated with pravastatin and captopril: ACE in atheromas provides cellular targets for captopril. *J. Cardiovasc. Pharmacol.,* **32,** 29–38.

[12] Kowala, M. C., Recce, R., Beyer, S., and Aberg, G. (1995) Regression of early atherosclerosis in hyperlipidemic hamsters induced by fosinopril and captopril. *J. Cardiovasc. Pharmacol.,* **25,** 179–186.

[13] Kowala, M. C., Grove, R. I., and Aberg, G. (1994) Inhibitors of angiotensin converting enzyme decreases early atherosclerosis in hyperlipidemic hamsters. Fosinopril reduces plasma cholesterol and captopril inhibits macrophage-foam cell accumulation independently of blood pressure and plasma lipids. *Atherosclerosis,* **108,** 61–72.

[14] Sharabi, Y., Grossman, E., Sherer, Y., Shaish, A., Levkovitz, H., Bitzur, R., and Harats, D. (2000) The effect of renin angiotensin axis inhibition on early atherogenesis in LDL receptor-deficient mice. *Pathobiology,* **68,** 270–274.

[15] Chobanian, A. V., Hope, S., and Brecher, P. (1995) Dissociation between the antiatherosclerotic effect of trandopril and suppression of serum and aortic angiotensin converting enzyme activity in Watanabe heritable hyperlipidemic rabbits. *Hypertension,* **25,** 1306–1310.

[16] Keren, P., George, J., Shaish, A., Levkovitz, H., Janakovic, Z., Afek, A., Goldberg, I., Kopolovic, J., Keren, G., and Harats, D. (2000) Effect of hyperglycemia and hyperlipidemia on atherosclerosis in LDL receptor deficient mice. *Diabetes,* **49,** 1064–1069.

[17] Knowles, J. W., and Maeda, N. (2000) Genetic modifiers of atherosclerosis in mice. *Atheroscler. Thromb. Vasc. Biol.,* **20,** 2336–2345.

[18] Kugiyama, K., Sugiyama, S., Matsumura, T., Ohta, Y., Doi, H., and Yasue, H. (1996) Suppression of atherosclerotic changes in cholesterol fed rabbits treated with an oral inhibitor of neutral endopeptidase 21.11(EC 3.4.24.11). *Atheroscler. Thromb. Vasc. Biol.,* **16,** 1080–1087.

[19] Grantham, J. A., Schirger, J. A., Wennberg, P. W., Sandberg, S., Heublein, D. M., Subkowski, T., and Burnnet, J. C. (2000) Modulation of functionally active endothelin converting enzyme by chronic neutral endopeptidase inhibition in experimental atherosclerosis. *Circulation,* **101,** 1976–1981.

[20] Tummala, P. E., Chen, X. L., Sundell, C. L., Laursen, J. B. L., Hammes, P., Alexander, W., Harrison, D. G., and Medford, R. M. (1999) Angiotensin II induces vascular cell adhesion molecule-1 expression in rat vasculature. *Circulation,* **100,** 1223–1229.

[21] Chen, X. L., Tummala, P. E., Olbrych, M. T., Alexander, R. W., and Medford, R. M. (1998) Angiotensin II induces monocyte chemoattractant protein-1 gene expression in rat vacular smooth muscle cells. *Circ. Res.,* **83,** 952–959.

[22] Grindling, K. K., Minieri, C. A., Ollerenshaw, J. D., and Alexander, R. W. (1994) Angiotensin II stimulates NADH and NADPH oxidase activation in cultured vascular smooth muscle cells. *Circ. Res.,* **74,** 1141–1148.

[23] Dimmeler, S., Rippmann, V., Weiland, U., Haendeler, J., and Zeiher, A. M. (1997) Angiotensin II induces apoptosis of human endothelial cells. Protective effect of nitric oxide. *Circ. Res.,* **81,** 970–976.

[24] Hope, S., Brecher, P., and Chobanian, A. V. (1999) Comparison of the effects of AT1 receptor blockade and angiotensin converting enzyme inhibition on atherosclerosis. *Am. J. Hypertens.,* **12,** 28–34.

[25] Rouleau, J. L., Pfeffer, M. A., Stewart, D. J., Issac, D., Sestier, F., Kerut, E. K., Porter, C. B., Proulx, G., Qian, C., and Block, A. J. (2000) Comparison of vasopeptidase inhibitor, omapatrilat, and lisinopril on exercise tolerance and mortality in patients with heart failure: IMPRESS randomised trial. *Lancet,* **356,** 615–620.

